# A Review on Plant Bioactive Compounds and Their Modes of Action Against Coronavirus Infection

**DOI:** 10.3389/fphar.2020.589044

**Published:** 2021-01-11

**Authors:** Juwairiah Remali, Wan Mohd Aizat

**Affiliations:** Institute of Systems Biology (INBIOSIS), Universiti Kebangsaan Malaysia (UKM), Bangi, Malaysia

**Keywords:** COVID-19, drug, herb, SARS, Traditional Chinese medicine (TCM), medicinal plant, natural products, viral infection

## Abstract

The rapid outbreak of coronavirus disease 2019 (COVID-19) has demonstrated the need for development of new vaccine candidates and therapeutic drugs to fight against the underlying virus, severe acute respiratory syndrome-coronavirus-2 (SARS-CoV-2). Currently, no antiviral treatment is available to treat COVID-19 as treatment is mostly directed to only relieving the symptoms. Retrospectively, herbal medicinal plants have been used for thousands of years as a medicinal alternative including for the treatment of various viral illnesses. However, a comprehensive description using various medicinal plants in treating coronavirus infection has not to date been described adequately, especially their modes of action. Most other reports and reviews have also only focused on selected ethnobotanical herbs such as Traditional Chinese Medicine, yet more plants can be considered to enrich the source of the anti-viral compounds. In this review, we have screened and identified potential herbal medicinal plants as anti-coronavirus medication across major literature databases without being limited to any regions or ethnobotanic criteria. As such we have successfully gathered experimentally validated *in vivo*, *in vitro,* or *in silico* findings of more than 30 plants in which these plant extracts or their related compounds, such as those of *Artemisia annua* L., *Houttuynia cordata* Thunb.*,* and *Sambucus formosana* Nakai, are described through their respective modes of action against specific mechanisms or pathways during the viral infection. This includes inhibition of viral attachment and penetration, inhibition of viral RNA and protein synthesis, inhibition of viral key proteins such as 3-chymotrypsin-like cysteine protease (3CL^pro^) and papain-like protease 2 (PL^pro^), as well as other mechanisms including inhibition of the viral release and enhanced host immunity. We hope this compilation will help researchers and clinicians to identify the source of appropriate anti-viral drugs from plants in combating COVID-19 and, ultimately, save millions of affected human lives.

## Introduction

Coronaviruses are known to infect various hosts such as mice (mouse hepatitis virus, MHV), pigs (porcine epidemic diarrhea virus, PEDV), birds (avian coronavirus, IBV) and even human (human coronavirus, HCoV including severe acute respiratory syndrome coronavirus (SARS-CoV), SARS-CoV-2, Middle East Respiratory Syndrome-CoV (MERS-CoV), HCoV-OC43, HCoV-NL63 and HCoV-229E) with different disease severity (Vellingiri et al., 2020; Zhu et al., 2020). On January 30, 2020, the World Health Organization (WHO) declared the outbreak of COVID-19, caused by the SARS-CoV-2, to be a global pandemic, which requires an international public health emergency (Patel and Jernigan, 2020). Ever since its outbreak in December 2019 (Yang et al., 2020), the SARS-CoV-2 virus has spread and caused more than 15.3 million cases and 631,000 deaths worldwide as of July 23, 2020 (https://virusncov.com/). The outbreak originated from the Hunan seafood market in Wuhan, a main city of China which frequently sold live exotic animals such as bats, frogs, snakes, dogs, civets, and marmots (Wang et al., 2020a). Although the zoonotic source of COVID-19 is yet to be verified, however, sequence-based analysis of isolates from infected patients has indicated that bats may serve as the primary reservoir, of which over 80% of the viral genome sequences is identical to the previous human SARS-coronavirus (Wu et al., 2020). Most COVID-19 patients initially suffered from fever, cough, and fatigue while developing other symptoms including muscle pain, headache, shortness of breath, and diarrhea, of which, in extreme cases, severe inflammatory responses may lead to fatality (Chen et al., 2020).

**FIGURE 1:**
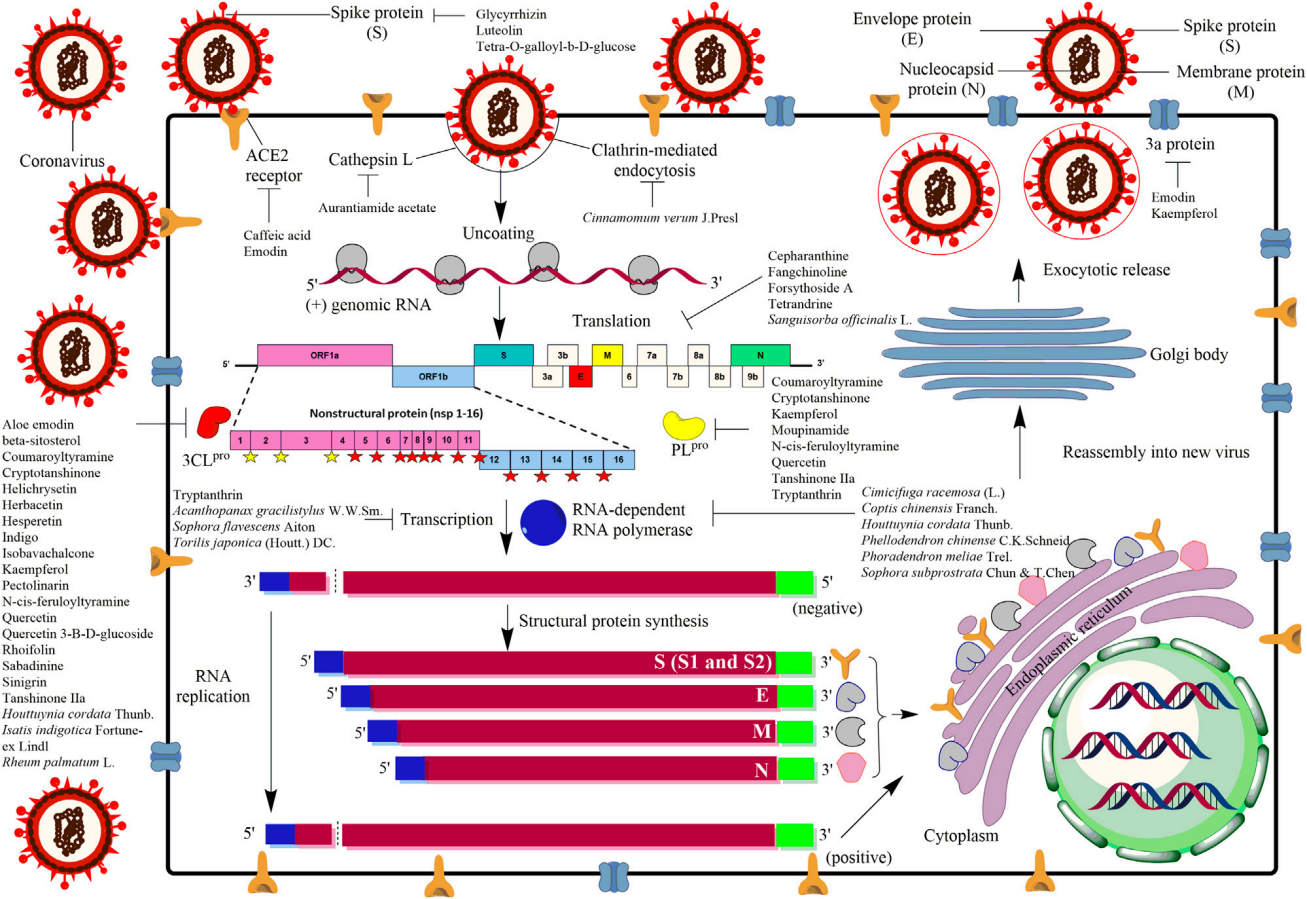
Mechanism of actions of antiviral agents/compounds from plants against coronavirus infection. As the virus initially infects an eukaryotic cell, several stages in the infection process occur which can be the potential sites of attack by the antiviral compounds. The life cycle of the virus begins when spike protein (S) of coronavirus binds to the angiotensin-converting enzyme 2 receptor (ACE2) located on the membrane of the host cells. The receptor binding facilitates viral envelope fusion with the host cell membrane before releasing the viral RNA into the host cell. The viral RNA encodes four structural proteins and two large non-structural polyproteins called ORF1a and ORF1b. These two non-structural polyproteins are translated and cleaved into 16 non-structural proteins (nsp) by two proteinases, 3-chymotrypsin-like cysteine protease (3CL^pro^) and papain-like protease 2 (PL^pro^). The yellow filled stars are the sites predicted to be cleaved by PLpro whereas the red filled stars are the sites predicted to be cleaved by 3CL^pro^. Besides that, the coronavirus genome also encodes structural proteins, including spike (S), envelope (E), membrane (M), and nucleocapsid (N) proteins. After replication, transcription and translation, viral proteins and genome RNA are subsequently assembled into virions in the endoplasmic reticulum (ER) and Golgi body before they will be transported via vesicles to be released out of the cell as new virus progenies.

SARS-CoV-2 belongs to the order *Nidovirales*, family *Coronaviridae* and genus *Coronavirus*, which is comprised of single stranded positive RNA sense viruses with 29.7 kb in length (Ksiazek et al., 2003; Marra et al., 2003; Ruan et al., 2003). Such RNA encodes two large non-structural polyproteins and four major structural proteins (Figure 1). The non-structural polyproteins, known as ORF1a and ORF1b with protein sizes of 486 and 790 kDa, respectively (Ksiazek et al., 2003; Marra et al., 2003), will undergo co-translational processing by two viral-encoded proteases called 3-chymotrypsin-like cysteine protease (3CL^pro^) and a papain-like protease (PL^pro^) (Gao et al., 2003; Snijder et al., 2003; Thiel et al., 2003). The processing of ORF1a is carried out by PL^pro^ at three sites (yellow filled star) which release nsp1, nsp2, and nsp3, whereas the rest of the sites are processed by 3CL^pro^ (red filled star) releasing nsp4 until nsp16 (Lindner et al., 2005). Besides that, the viral RNA encodes for the structural proteins, including nucleocapsid (N), envelope (E), membrane (M), and spike (S) proteins (Figure 1) (Du et al., 2016; Zhou et al., 2018; Wang et al., 2020b). The N protein is a soluble protein that contains the nucleocapsid packages of the RNA genome. Whereas, E is a small protein, around 76–109 amino acids containing a single predicted hydrophobic domain that is essential for interaction with M protein (Ruch and Machamer, 2012) which is the most prevalent membrane protein in the virion envelope (Masters, 2006; Hogue and Machamer, 2014). Meanwhile, the S protein serves as an important mechanism for viral attachment and fusion to the host cells (Figure 1). In general, upon S protein attachment to the host receptor called angiotensin-converting enzyme 2 (ACE2), the coronavirus will enter into the host cell through endocytosis (Figure 1). The virus will then release the genomic RNA before the genetic codes are being translated into non-structural polyproteins including 3CL^pro^, PL^pro^ and RNA-dependent RNA polymerase (RdRp), followed by other structural proteins (N, M, E and S) (Kumar et al., 2020). Eventually, the viral progenies will bud out with all the complete components including replicated genomic RNA to infect other neighboring regions (Figure 1).

Due to its infectivity, scientists have been racing to comprehend the origin and transmission of the virus including elucidating the mechanism for its disease pathogenesis (Liu et al., 2020). A number of comprehensive research have unraveled viable viral genes and proteins as promising future targets for the development of therapeutic agents and vaccines for COVID-19 or other related human coronaviruses (Guo et al., 2020; Liu et al., 2020). However, there are still no clinically approved vaccine or specific antiviral drugs to fight the infection as of July 2020, although more than nine pharmaceutical companies are currently working on this disease (https://www.forbes.com/sites/moneyshow/2020/06/16/9-pharmaceutical-companies-racing-for-a-covid-19-vaccine/#3acd13c576ad). Current treatments used by physicians mainly depend on managing the symptoms such as breathing support using mechanical ventilation, and corticosteroids to reduce lung swelling, as well as the current available antiviral and antibiotics medications (Cascella et al., 2020).

Alternatively, herbal medicine has been used for thousands of years to treat various viral-related illnesses and may become a valuable source of anti-coronovirus treatment (Ling, 2020). Evidently, in 2003, patients who suffered from severe acute respiratory syndrome (SARS) and received Traditional Chinese Medicine (TCM) treatment were reported to have short-term hospitalization (inpatient care), reduced steroid-related side effects, and improvement from the viral symptoms (World Health Organization, 2004). Thus, most previous publications including review papers have mainly focused on the use of TCM and other ethnobotanical herbs (Dudani and Saraogi, 2020; Khanna et al., 2020; Ling, 2020; Vellingiri et al., 2020) or the use of drugs and other dietary supplements that are currently in clinical trials (Di Matteo et al., 2020; Kumar et al., 2020; Stahlmann and Lode, 2020). This may not cover most, if not all, potential plant species or extracts available globally. Besides that, the mechanism of these plant extracts and compounds, especially their modes of action against the viral infection, are still not comprehensively described (Dudani and Saraogi, 2020; Ling, 2020). Therefore, in this review, we have gathered previous studies on medicinal herbs with some evidence against coronavirus infection, either through *in vivo*, *in vitro,* or *in silico* studies, and classified them based on their different modes of actions. This review highlights the potential use of herbal plants and related compounds in treating the coronavirus infection, in the hope that it could lead to a potential source of anti-viral drugs for curbing the COVID-19 outbreak.

All manuscripts were obtained from various databases such as Scopus, PubMed, and Web of Science by using key words (herb* OR herbal) AND (virus OR viral) AND corona* in the search field. We have identified and included research studies starting from year 2004 to year 2020. Relevant papers were selected after critical evaluation on their significance and were further divided based on their experimental designs, whether *in vitro*, *in vivo,* or *in silico* (Tables 1–3). However, we acknowledge that the recorded assay values, for examples IC50 and SI values from the *in vitro* analysis (Table 1), are not comparable between cell types/lines nor coronavirus types (Kalliokoski et al., 2013) due to their inherent molecular and physiological differences. Thus, the plant herbal extracts/compounds are only discussed within specific application (cell and virus types) of their original reported studies, to avoid over interpretation. Furthermore, the potential use of various herbs are described in this review according to their modes of action including inhibition of viral attachment and penetration, inhibition of RNA and protein synthesis, inhibition of viral proteases, inhibition of viral release and enhancement of host immunity as well as other mechanisms.

**TABLE 1:** *In vitro* antiviral activity of various herbal extracts against coronavirus infection.

No.	Herbs	Extract/Compound	Coronavirus type	CC50 (conc.) or cell survivability (%)	IC50	SI	References
1.	*Acanthopanax gracilistylus* W.W.Sm.	Methanol extract	MHV-A59	170.0 ± 6.4 μg/ml	0.9 ± 0.1 μg/ml	188.9	Kim et al. (2010)
2.	*Artemisia annua* L.	Ethanol extract	SARS-CoV BJ-001	1,053.0 ± 92.8 μg/ml	34*.*5 ± 2.6 μg/ml	31	Li et al. (2005)
			SARS-CoV BJ-006	1,053.0 ± 92.8 μg/ml	39*.*2 ± 4.1 μg/ml	27	
3.	*Cimicifuga racemose* (L.) Nutt.	Methanol extract	MHV-A59	239.0 ± 44.4 μg/ml	19.4 ± 7.0 μg/ml	12.3	Kim et al. (2008)
4.	*Cinnamomum verum* J.Presl (cortex)	Butanol extract	wtSARS-CoV	180.0 ± 6.0 μg/ml	7.8 ± 0.3 μg/ml	23.1	Zhuang et al. (2009)
			HIV/SARS-CoV	444.0 ± 13.7 μg/ml	85.3 ± 7.5 μg/ml	5.2	
		Procyanidin A2	wtSARS-CoV	1,116.7 ± 60.3 µM	29.9 ± 3.3 µM	37.35	
			HIV/SARS-CoV	796.6 ± 63.7 µM	120.7 ± 13.1 µM	4.08	
		Procyanidin B1	wtSARS-CoV	648.2 ± 43.4 µM	41.3 ± 3.4 µM	15.69	
			HIV/SARS-CoV	656.2 ± 36.7 µM	161.1 ± 20.3 µM	4.08	
		Cinnamtannin B1	wtSARS-CoV	184.7 ± 15.5 µM	32.9 ± 3.9 µM	5.61	
			HIV/SARS-CoV	242.3 ± 14.8 µM	32.9 ± 2.8 µM	7.36	
5.	*Coptis chinensis* Franch.	Methanol extract	MHV-A59	71.3 ± 7.2 μg/ml	2.0 ± 0.5 μg/ml	34.9	Kim et al. (2008)
6.	*Epimedium koreanum* Nakai	Water extract	PEDV, SM98, TGE	ND	>90% at 1.5 mg/ml	ND	Cho et al. (2012)
7.	*Euphorbia neriifolia* L.	3β-friedelanol	HCoV	NC	132.4% at 5 μg/ml	NC	Chang et al. (2012)
		3β-acetoxy friedelane	HCoV	NC	80.9% at 5 μg/ml	NC	
		Friedelin	HCoV	NC	109.0% at 5 μg/ml	NC	
		Epitaraxerol	HCoV	NC	111.0% at 5 μg/ml	NC	
8.	*Forsythia suspensa* (Thunb.) Vahl	Forsythoside A	IBV	<1.28 mM	0.64 mM	ND	Li et al. (2011)
9.	*Glycyrrhiza glabra* L.	Glycyrrhizin	SARS-CoV	>20,000 mg/ml	300 mg/ml	>67	Cinatl et al. (2003), Hoever et al. (2005), Hoever et al. (2005)
		Glycyrrhizin	SARS-CoV	>24,000 μM	365 ± 12 μM	>65	
		Glycyrrhetinic acid	SARS-CoV	>20 μM	20 ± 5 μM	NC	
		Derivative GL 1	SARS-CoV	>3,000 μM	40 ± 13 μM	>75	
		Derivative GL 2	SARS-CoV	1,462 ± 50 μM	35 ± 7 μM	41	
		Derivative GL 3	SARS-CoV	215 ± 18 μM	139 ± 20 μM	2	
		Derivative GL 9	SARS-CoV	44 ± 6 μM	8 ± 2 μM	6	
		Derivative GL 10	SARS-CoV	250 ± 19 μM	50 ± 10 μM	5	
		Derivative GL 11	SARS-CoV	15 ± 3 μM	5 ± 3 μM	3	
		Derivative GL 12	SARS-CoV	66 ± 8 μM	16 ± 1 μM	4	
10.	*Houttuynia cordata* Thunb.	Water extract	IBV	250 mg/ml	62.5 mg/ml	4	Yin et al. (2011)
		Water extract	SARS-CoV	NC	1,000 μg/ml	NC	Lau et al. (2008)
		Water extract	SARS-CoV	NC	50, 100, 200, 400 and 800 μg/ml	NC	Lau et al. (2008)
		Ethyl acetate	MHV	>3.91 μg/ml	0.98 μg/ml	>4.00	Chiow et al. (2016)
		Quercetin	MHV	116.52 μg/ml	125 μg/ml	0.93	Chiow et al. (2016)
11.	*Isatis indigotica* Fortune ex Lindl.	Water extract	SARS-CoV	>5,000 μg/ml	191.6 ± 8.2 μg/ml	>26	Lin et al. (2005)
		Indigo	SARS-CoV	7,375 μM	752 μM	9.8	
		Sinigrin	SARS-CoV	>10,000 μM	217 μM	>46	
		Beta-sitosterol	SARS-CoV	1,475 μM	1,210 μM	1.21	
		Aloe emodin	SARS-CoV	11,592 μM	366 μM	31.67	
		Hesperetin	SARS-CoV	2,718 μM	8.3 μM	327.47	
12.	*Lindera aggregata* (Sims) Kosterm.	Ethanol extract	SARS-CoV BJ-001	1,374.0 ± 39.0 μg/ml	88*.*2 ± 7.7 μg/ml	16	Li et al. (2005)
			SARS-CoV BJ-006	1,374.0 ± 39.0 μg/ml	80*.*6 ± 5.2 μg/ml	17	
13.	*Lycoris radiata* (L'Hér.) Herb.	Ethanol extract	SARS-CoV BJ-001	886.6 ± 35.0 μg/ml	2*.*4 ± 0.2 μg/ml	370	Li et al. (2005)
			SARS-CoV BJ-006	886.6 ± 35.0 μg/ml	2*.*1 ± 0.2 μg/ml	422	
		Lycorine	SARS-CoV	14,980 nM	15.7 nM	954.14	
14.	*Phoradendron meliae* Trel.	Methanol extract	MHV-A59	334.3 ± 7.0 μg/ml	13.0 ± 1.4 μg/ml	25.6	Kim et al. (2008)
15.	*Pelargonium sidoides* DC.	Aqueous extract (EPs® 7630)	HCoV-229E	>100 μg/ml (87%)	44.5 μg/ml	>2.3	Michaelis et al. (2011)
16.	*Phellodendron chinense* C.K.Schneid.	Methanol extract	MHV-A59	139.5 ± 81.3 μg/ml	10.4 ± 2.2 μg/ml	13.4	Kim et al. (2008)
17.	*Polygonum multiflorum* Thunb. (vine)	Water extract	SARS-CoV	NC	1–10 μg/ml	NC	Ho et al. (2007)
	*Polygonum multiflorum* Thunb. (root)	Emodin	SARS-CoV	NC	200 μM	NC	Ho et al. (2007)
		Water extract	SARS-CoV	NC	1–10 μg/ml	NC	Ho et al. (2007)
18.	*Pyrrosia lingua* (Thunb.) Farw.	Chloroform	SARS-CoV BJ-001	2,378.0 ± 87.3 μg/ml	43*.*2 ± 14.1 μg/ml	55	Li et al. (2005)
			SARS-CoV BJ-006	2,378.0 ± 87.3 μg/ml	40*.*5 ± 3.7 μg/ml	59	
19.	*Rheum officinale* Baill. (root)	Water extract/Emodin	SARS-CoV	NC	1–10 μg/ml	NC	Ho et al. (2007)
20.	*Rheum palmatum* L.	Ethyl acetate extract	SARS-CoV	NC	13.76 ± 0.03 μg/ml	NC	Luo et al. (2009)
21.	*Rhus chinensis* Mill.	Tetra-O-galloyl-β-d-glucose (TGG)	SARS-CoV	1,080 μM	4.5 μM	240	Wu et al. (2004)
22.	*Sanguisorba officinalis* L.	Methanol extract	MHV-A59	388.4 ± 4.5 μg/ml	3.7 ± 1.4 μg/ml	105.0	Kim et al. (2010)
23.	*Sambucus formosana* Nakai	Ethanol extract	HCoV-NL63	180.62 ± 63.04 μg/ml	1.17 ± 0.75 μg/ml	154.37	Weng et al. (2019)
		Caffeic acid	HCoV-NL63	>500 μM	3.54 ± 0.77 μM	NC	
		Chlorogenic acid	HCoV-NL63	>500 μM	43.45 ± 6.01 μM	NC	
		Gallic acid	HCoV-NL63	>500 μM	71.48 ± 18.40 μM	NC	
24.	*Sophora flavescens* Aiton	Methanol extract	MHV-A59	556.8 ± 2.9 μg/ml	0.8 ± 0.2 μg/ml	696.0	Kim et al. (2010)
25.	*Sophora subprostrata* Chun & T.Chen	Methanol extract	MHV-A59	307.3 ± 6.6 μg/ml	27.5 ± 1.1 μg/ml	11.1	Kim et al. (2008)
26.	*Stephania tetrandra* S.Moore	Tetrandrine	HCoV-OC43	13.41 ± 0.36 μM	0.33 ± 0.03 μM	40.19	Kim et al. (2019)
		Fangchinoline	HCoV-OC43	11.54 ± 0.46 μM	1.01 ± 0.07 μM	11.46	
		Cepharanthine	HCoV-OC43	11.26 ± 0.69 μM	0.83 ± 0.07 μM	11.63	
27.	*Strobilanthes cusia* (Nees) Kuntze	Methanol extract	HCoV-NL63	>100 μg/ml	0.64 ± 0.43 μg/ml	156.25	Tsai et al. (2020)
		Tryptanthrin	HCoV-NL63	>400 μM	1.52 ± 0.13 μM	263.16	
		Indigodole B	HCoV-NL63	>400 μM	2.60 ± 0.11 μM	153.85	
28.	*Toona sinensis* (Juss.) M.Roem.	Water extract	SARS-CoV	>500 μg/ml	30–43 μg/ml	17	Chen et al. (2008)
29.	*Torilis japonica* (Houtt.) DC.	Methanol extract	MHV-A59	156.5 ± 2.6 μg/ml	0.8 ± 0.0 μg/ml	195.6	Kim et al. (2010)
30.	*Veronica linariifolia* Pall. ex Link	Luteolin	SARS-CoV	10,600 μM	10.6 μM	14.62	Wu et al. (2004)
31.	Various (Chinese medicinal herbs)	Emodin	SARS-CoV	NC	50% at 20 μM	NC	Schwarz et al. (2012)
		Kaempferol	SARS-CoV	NC	20% at 20 μM	NC	
		Kaempferol glycosides	SARS-CoV	NC	>50% at 20 μM	NC	
32.	Various (used flavonoid library)	Herbacetin	MERS & SARS-CoV	NC	33.17–40.59 μM	NC	Jo et al. (2019), Jo et al. (2020)
		Rhoifolin	SARS-CoV	NC	27.45 μM	NC	Jo et al. (2020)
		Pectolinarin	SARS-CoV	NC	37.78 μM	NC	Jo et al. (2020)
		Isobavachalcone	MERS-CoV	NC	35.85 μM	NC	Jo et al. (2019)
		Quercetin 3-β-d glucoside	MERS-CoV	NC	37.03 μM	NC	Jo et al. (2019)
		Helichrysetin	MERS-CoV	NC	67.04 μM	NC	Jo et al. (2019)

CC50 is the cytotoxicity concentration of the extracts or compounds that can inhibit 50% population of host cells. IC50 is the inhibitory concentration of the extracts or compound that can cause 50% of virus inhibition or reduction after it has been treated with extract/compound. Selectivity index (SI) is calculated based on the ratio of CC50/IC50. The higher the value of SI, the lesser cytotoxicity for the host and hence being potentially safe to be applied as a future antiviral agent. NC is non-calculable, and ND is data not determined/shown from respective articles.

Abbreviation: MHV, mouse hepatitis virus; wtSARS-CoV; wild-type SARS coronavirus; HIV, human immunodeficiency virus; PEDV, porcine epidemic diarrhea virus; TGE, transmissible gastroenteritis coronavirus; HCoV, human coronavirus; IBV, avian coronavirus; 3CL^pro^, 3-chymotrypsin-like cysteine protease; PL^pro^, papain-like protease 2; RdRp, RNA-dependent RNA polymerase; SARS, severe acute respiratory syndrome; MERS, middle east respiratory syndrome

**TABLE 2:** *In silico* antiviral activity of various herbal extracts against coronavirus protein target.

No.	Herbs	Compounds	Inhibition target	Docking binding energy (kcal/mol)		References
				PLpro	3CLpro	
1.	Various (Traditional Chinese Medicine Systems Pharmacology Database)	Coumaroyltyramine	PL^pro^ and 3CL^pro^	−3.22	−4.18	Zhang et al. (2020)
		Cryptotanshinone	PL^pro^ and 3CL^pro^	−5.25	−6.23	
		Desmethoxyreserpine	Replication, 3CL^pro^, 3CL^pro^	Not significant	−3.52	
		Dihomo-γ-linolenic acid	S protein	Not significant	−3.88	
		Kaempferol	Replication, 3CL^pro^	−2.15	−6.01	
		Lignan	PL^pro^	Not significant	−4.27	
		Moupinamide	PL^pro^ and 3CL^pro^	−3.05	Not significant	
		N-cis-feruloyltyramine	PL^pro^ and 3CL^pro^	−3.11	−4.31	
		Quercetin	Replication, 3CL^pro^	−4.62	−6.25	
		Sugiol	PL^pro^ and 3CL^pro^	Not significant	−6.04	
		Tanshinone IIa	PL^pro^ and 3CL^pro^	−5.02	−5.17	
2.	Various (Traditional Chinese Medicine Database)	Aurantiamide acetate	SARS-CoV cathepsin-L (−50.767 kcal/mol)	Not carried out	Not carried out	Wang et al. (2007)
3.	Various (flavonoid library)	Herbacetin	SARS & MERS-CoV 3CL^pro^	Not carried out	–9.263 & −10.246	Jo et al. (2020)
		Rhoifolin	SARS-CoV 3CL^pro^	Not carried out	–9.565	
		Pectolinarin	SARS-CoV 3CL^pro^	Not carried out	–8.054	
		Isobavachalcone	MERS-CoV 3CL^pro^	Not carried out	−9.364	Jo et al. (2019)
		Quercetin 3-β-d glucoside	MERS-CoV 3CL^pro^	Not carried out	−9.751	
		Helichrysetin	MERS-CoV 3CLpro	Not carried out	−9.953	
4.	*Veratrum sabadilla* Retz.	Sabadinine	3CL^pro^	Not carried out	−11.6	Toney et al. (2004)

**TABLE 3:** *In vivo* antiviral activity of various herbal extracts against coronavirus infection.

No.	Herbs	Extract/Compound	Coronavirus type	Animal model	Concentration	Observation, survivability and/or biopsy	References
1.	*Allium sativum* L.	Water extract	IBV	Chicken embryo	0.1 ml of extract	Less dwarfism observed in chicken embryos treated with garlic	Mohajer Shojai et al. (2016)
2.	*Epimedium koreanum* Nakai	Water extract	PEDV	Pig	0.6% of extract	No disease symptoms such as diarrhoea	Cho et al. (2012)
						Biopsy results showed clean intestine	
3.	*Houttuynia cordata* Thunb.	Water extract	IBV	Chicken embryo	62.5 mg/ml	Fully protected the chicken embryos and more than 50% protection rate in chickens	Yin et al. (2011)

### Inhibition of Viral Attachment and Penetration

The inhibition of viral penetration and attachment is an effective way to curb coronavirus infection. Infectious virion binds to cell membrane receptors, permeates the cellular membranes and removes the virion's protein coat once entered into the cell cytoplasm, releasing viral nucleic acid (Figure 1) (Smith et al., 1980). Coronavirus S protein plays a critical role in viral attachment, fusion, and entry, making it a potential target in the development of vaccines, antibodies, and inhibitors (Liu et al., 2004; Du et al., 2009, 2017; Wang et al., 2016). The S protein modulates the viral penetration through the first binding on the host cells using receptor-binding domain in the S1 subunit and then fusion into host cells via the S2 subunit through the host ACE2 receptor (Liu et al., 2004). However, different subtypes of coronavirus have been described to recognize different receptors. For examples, SARS-CoV specifically recognizes the ACE2 receptors, while MERS-CoV identified dipeptidyl peptidase 4 (DPP4) receptors (Li et al., 2003; Raj et al., 2013). The current SARS-CoV-2 binds ACE2 receptor (Zhou et al., 2020), thus inhibition at these S proteins or ACE2 may inhibit the viral attachment from entering host cells (Figure 1).

**FIGURE 2:**
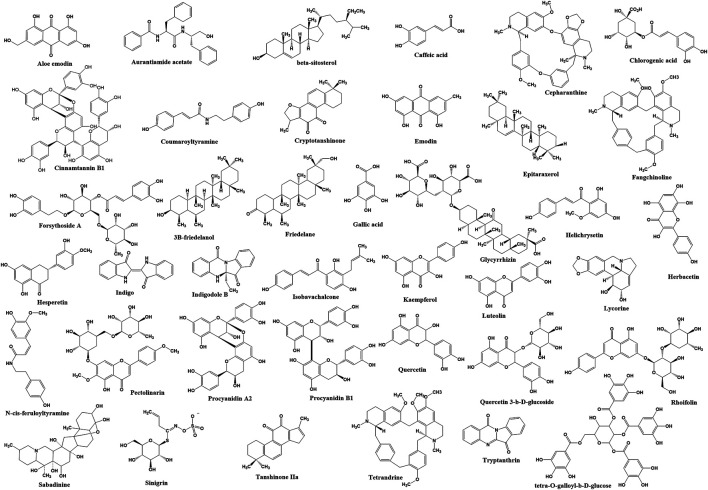
The chemical structure of bioactive compounds identified from plants that have inhibition effects on coronavirus infection.

Among 121 herbal compounds that were screened by Wu et al. (2004), tetra-O-galloyl-β-d-glucose (TGG) and luteolin, isolated from *Rhus chinensis* Mill. and *Veronica linariifolia* Pall. ex Link, respectively, were identified to have the highest affinity to the S2 subunit of the virus, thus postulating that these compounds may interfere with the viral cell fusion process (Figures 1, 2) (Wu et al., 2004). TGG and luteolin exhibit significant anti-SARS-CoV activity with IC_50_ of 4.5 and 10.6 µM, respectively, as well as very high selectivity index of 240.0 and 14.62, respectively (Table 1). Selectivity index (SI) is determined based on the proportion of cytotoxicity concentration of the extracts or compounds that can inhibit 50% population of host cells (CC_50_) to inhibitory concentration of the extracts or compounds that can cause 50% of virus inhibition (IC_50_). The higher the value of SI, the lesser cytotoxicity for the host and hence it being potentially safe to be applied as a future antiviral agent (Table 1 and Figure 2) (Wu et al., 2004). Thus, this suggested that both compounds can be effective against the coronavirus and, more importantly, safe for human application.

In addition, glycyrrhizin isolated from *Glycyrrhiza glabra* L. (licorice) also has been identified to inhibit viral attachment and penetration (Figure 1) (Cinatl et al., 2003). In addition, licorice root has been known historically as a powerful antiviral herb (Fiore et al., 2008), used thousands of years ago as a folk medicine for the treatment of throat infection, asthma, bronchitis, peptic ulcers, inflammation, and allergies (Das et al., 1989; Krausse et al., 2004; Nassiri Asl and Hosseinzadeh, 2008). Glycyrrhizin or its other name glycyrrhizic acid (Figure 2) is the principal bioactive component of triterpenoid glycoside in this herb, having demonstrated promising inhibition of SARS-CoV (Chen et al., 2004). Evidently, the compound was found more effective (SI = over 67; Table 1) against the replication of clinical isolates of SARS-CoV than several synthetic antivirals, such as ribavirin, pyrazofurin, 6-azauridine, and mycophenolic acid (Cinatl et al., 2003). Glycyrrhizin also has been shown to suppress inflammation through downregulation of proinflammatory mediators (Ramos-Tovar and Muriel, 2019; Khanna et al., 2020), but its effectiveness in combating COVID-19 severe inflammation including cytokine storm requires further investigation.

Another study conducted on 312 controlled traditional herbs successfully identified two species from the family *Polygonaceae* to inhibit the interaction of coronavirus S protein and ACE2 (Ho et al., 2007). Water extracts from the root tubers of *Rheum officinale* Baill*.*, as well as the vines and root tubers of *Polygonum multiflorum* Thunb., showed IC_50_ range of 1–10 μg/ml for the viral inhibition (Table 1) (Ho et al., 2007). Emodin, an anthraquinone glycoside compound found from both species (Figure 2), significantly obstructed the S protein and ACE2 interaction (Figure 1) in a dose-dependent way (Ho et al., 2007). This compound also inhibited the S protein-pseudotyped retrovirus infectivity in Vero E6 cells (Ho et al., 2007), thus showing promising potential for blocking the viral entry (Figure 1) (Ho et al., 2007).

Besides that, ethanol extract of *Sambucus formosana* Nakai, a species of elderberry, also has been identified to have very potent anti-viral effect as it has very low IC50 at 1.17 μg/ml and high SI of 154.37, against the coronavirus (Table 1) (Weng et al., 2019). Caffeic acid isolated from the plant extract (Figure 2) also demonstrated potential anti-coronavirus activity through viral entry inhibition. This compound was able to impair the binding interaction of human coronavirus NL63 (HCoV-NL63) with ACE2 receptor (Figure 1) (Chiou et al., 2017) and heparan sulphate proteoglycans (co-receptor) (Milewska et al., 2014) on the host cell surface. In addition to caffeic acid, two other phenolic acid constituents from this plant extract, chlorogenic acid and gallic acid, were found to exhibit antiviral effect by reducing the development of HCoV-NL63 particles *in vitro* (Table 1 and Figure 2) (Weng et al., 2019). The IC50 of these two compounds showed promising viral inhibition (chlorogenic acid IC50 = 43.45 μM and gallic acid IC50 = 71.48 μM) but the caffeic acid was more potent (IC50 = 3.54 μM) (Table 1) (Weng et al., 2019).

The coronavirus S-protein also utilizes endosomal cathepsin l-protease enzymatic activity for viral entry (Figure 1) (Simmons et al., 2005; Huang et al., 2006). Cathepsin L stimulates the S protein-mediated membrane fusion by promoting receptor dependent and acid-dependent conformational changes in the S2 domain in which a low pH permits optimal proteolytic activity in endosomes (Huang et al., 2006; Bosch et al., 2008). This indicates that the S protein may be triggered proteolytically during the entry of the virus into infected cells throughout the endocytic route (Simmons et al., 2005; Huang et al., 2006). Screening of various natural compounds based on the Traditional Chinese Medicine (TCM) database has identified aurantiamide acetate derived from *Artemisia annua* L. plant (Figure 2) (Wang et al., 2007) to inhibit the SARS cathepsin L with the lowest docking binding energy (−50.767 kcal/mol) (Table 2), thus possibly blocking viral entry (Figure 1) (Wang et al., 2007). This plant is originally known to exhibit many therapeutic efficacies especially against flu-like symptoms, such as antitracheitis (inflammation at trachea), cough relief, phlegm removal, asthma relief and others (Zhou et al., 2011). These findings suggest the compound could be implemented as medication to prevent SARS-CoV infection, although this should be evaluated further in animal studies in future (Du et al., 2009).

SARS-CoV also utilizes endocytosis route mediated by clathrin to invade host cells (Figure 1) (Inoue et al., 2007). Clathrin-based endocytosis has been well characterized to use growth factor receptors such as the transferrin receptor (TfR) (Mellman, 1996), epidermal growth factor receptor (Stang et al., 2004), and the keratinocyte growth factor receptor (Belleudi et al., 2003). Butanol fraction of *Cinnamomum verum* J. Presl (cortex) was shown to inhibit wild-type SARS-CoV (wtSARS-CoV) infection and HIV/SARS-CoV S pseudovirus infections by inhibiting clathrin-mediated endocytosis pathway through TfR receptor (Table 1 and Figure 1) (Zhuang et al., 2009). Besides that, procyanidin A2, procyanidin B1, and cinnamtannin B1 successfully fractionated from this plant (Figure 2) showed moderate anti-wtSARS-CoV activity (IC50s of 29.9, 41.3 and 32.9 µM, respectively, and SIs of 37.35, 15.69, and 5.61, respectively) (Table 1) (Zhuang et al., 2009). However, the procyanidins have not been shown to inhibit the internalization of the virus (Zhuang et al., 2009) and hence more studies needs to be performed to elucidate their exact mechanism of inhibition.

### Inhibition of RNA and Protein Synthesis

Several plants such as *Sanguisorba officinalis* L.*, Stephania tetrandra* S. Moore, and *Strobilanthes cusia* (Nees) Kuntze also have anti-viral activity towards RNA and protein synthesis of the coronavirus. For instance, N protein expression of mouse hepatitis virus, MHV-A59 was reduced in *Sanguisorba officinalis* L. treatment, suggesting this plant extract might negatively affect the viral nucleocapsid formation (Figure 1) (Kim et al., 2010). Besides that, natural bis-benzylisoquinoline alkaloids such as tetrandrine, fangchinoline, and cepharanthine isolated from *Stephania tetrandra* S.Moore (Figure 2) were able to suppress the expression of viral S and N proteins, thus inhibiting the replication of human coronavirus OC43 (HCoV-OC43) infection in MRC-5 human lung cells (Figure 1) (Kim et al., 2019). In addition to that, *Strobilanthes cusia* (Nees) Kuntze leaf methanol extract strongly suppressed HCoV-NL63-infected cells with IC50 of 0.64 μg/ml (Table 1), and the HCoV-NL63 infection was potently inhibited in a concentration-dependent manner (Tsai et al., 2020). Tryptanthrin and indigodole B (5aR-ethyltryptanthrin) are among the various bioactive compounds present in the extract of *S. cusia* (Nees) Kuntze (Figure 2) that exhibited significant antiviral activity in minimizing the cytopathic effect and development of viral progeny with IC_50_ values of 1.52 and 2.60 µM, respectively (Table 1) (Tsai et al., 2020). Different modes of time-of-addition/removal assay demonstrated that tryptanthrin prevented the replication of HCoV-NL63 in the early and the late replication stages, mainly by blocking genome synthesis of viral RNA (Figure 1) (Tsai et al., 2020).

Furthermore, intracellular viral RNA concentrations were also reduced in the extracts of *Sophora flavescens* Aiton*, Acanthopanax gracilistylus* W.W.Sm., and *Torilis japonica* (Houtt.) DC. with a comparable reduction in viral protein and MHV-A59 production (Kim et al., 2010). Moreover, the extracts reduced the replication of other subtypes of coronavirus such as the John Howard Mueller strain of MHV and porcine epidermic diarrhea virus (PEDV) *in vitro* (Kim et al., 2010). Besides that, an active ingredient of forsythoside A isolated from the fruits of *Forsythia suspensa* (Thunb.) Vahl has been identified to reduce the viral yield and decreased the expression of infectious bronchitis virus (IBV) N gene (Figure 1) in contrast to untreated IBV infected cells (Li et al., 2011). Forsythoside A is completely able to inhibit IBV in primary chicken embryo kidney cells and abrogated the virus progeny *in vitro* at concentration of 0.64 mM (Table 1 and Figure 2) (Li et al., 2011). This compound also effectively inhibited pathogenic bacteria including *Staphylococcus aureus* (Nishibe et al., 1982). *Forsythia suspensa* (Thunb.) Vahl is typically used to prevent inflammation, pyrexia and emesis in traditional Chinese medicine (Li et al., 2011).

RNA-dependent RNA polymerase (RdRp) is a replicase enzyme that is crucial for the transcription and replication of coronavirus (Thiel et al., 2003). Since RdRp has been identified to play a vitally important role for the virus life cycle, several polymerase inhibitors such as Remdesivir have been implemented for the treatment of varying viral infections such as human immunodeficiency virus type 1 (HIV-1), human hepatitis B virus (HBV), hepatitis C virus (HCV), Zika virus, and herpes virus (Korba et al., 2006; Mercorelli et al., 2018; Gordon et al., 2020). Thus, the inhibition of this enzyme can potentially be used for the discovery of anti-SARS-CoV agent (Lau et al., 2008). For example, *H. cordata* Thunb. has been identified to act on RdRp activity (Figure 1) (Lau et al., 2008). A study on the polymerase activity with different concentration levels of *H. cordata* Thunb. water extract (50, 100, 200, 400 and 800 μg/ml) showed a marked reduction in RdRp activity (Table 1) (Lau et al., 2008). Moreover, the oral acute toxicity test showed that *H. cordata* Thunb. was not harmful toward mice after implementing oral administration at 16 g/kg (Lau et al., 2008) and 2000 mg/kg (Chiow et al., 2016), thus it would be potentially safe for human consumption. Several plant extracts from *Cimicifuga racemosa* (L.) Nutt.*, Phellodendron chinense* C.K.Schneid., *Sophora subprostrata* Chun & T.Chen, *Phoradendron meliae* Trel*.,* and *Coptis chinensis* Franch. were also known to inhibit RdRp activity (Figure 1) (Kim et al., 2008). Methanol extracts from these plants decreased the production of intracellular viral RNA and protein expression in murine coronavirus (MHV) with IC_50_ values between 2.0 and 27.5 μg/ml (Table 1) (Kim et al., 2008). These extracts also significantly decreased PEDV production *in vitro,* a coronavirus that infects the cell lining of pig small intestine (Kim et al., 2008).

### Inhibition of Viral Proteases, 3CL^pro^ and PL^pro^

Viral proteases such as 3CL^pro^ and PL^pro^ also play a prominent role in the replication of coronavirus and presently have become the potential drug target for the development of anti-coronavirus agents (Anand et al., 2003; Han et al., 2005; Gan et al., 2006; Zhang et al., 2020). These two proteases (PL^pro^ and 3CL^pro^) are responsible for the synthesis and maturation of the various viral polyproteins as shown in Figure 1, and hence they are vital for the biogenesis of the virus replication complex (Lindner et al., 2005). We identified several plants and compounds that have significant activities to inhibit these enzymes. However, compared to PL^pro^, many more studies have been conducted on 3CL^pro^, possibly as it was able to generate 12 important non-structural proteins (nsp 4 to nsp 16), including the viral RdRp (nsp 12) and helicase (nsp 13) (Rut et al., 2020).

*In silico* docking analysis has identified 13 herbal compounds such as coumaroyltyramine, cryptotanshinone, kaempferol, N-cis-feruloyltyramine, quercetin, and tanshinone IIa that are able to inhibit 3CL^pro^ and PL^pro^ (Figures 1, 2 and Table 2) (Zhang et al., 2020). Furthermore, tryptanthrin isolated from the extract of *S. cusia* (Nees) Kuntze (Figure 2, Table 1) also has been identified to inhibit PL^pro^ activity of the HCoV-NL63 *in vitro* (Figure 1) (Tsai et al., 2020). Another *in silico* study has identified several other plant compounds such as helichrysetin, herbacetin, isobavachalcone, pectolinarin, quercetin 3-β-D-glucoside, and rhoifolin (Figure 2) to effectively interrupt the enzymatic activity of 3CL^pro^ of coronavirus (Figure 1 and Table 2) (Jo et al., 2019, 2020). Moreover, sabadinine, a naturally occurring bioactive compound originally isolated from the Lily plant *Veratrum sabadilla* Retz. (Figure 2) (Sayre, 1907), was also shown able to dock into the active site of 3CL^pro^ (Table 2) (Toney et al., 2004).

Besides that, *H. cordata* Thunb.*, Isatis indigotica* Fortune ex Lindl. and *R. palmatum* L. also exhibited significant inhibitory effects on 3CL^pro^ of SARS-CoV in *in vitro* experiments (Figure 1 and Table 1) (Lin et al., 2005; Lau et al., 2008; Luo et al., 2009). The activity of 3CL^pro^ was reduced to 50% at the maximum concentration of 1,000 μg/ml of *H. cordata* Thunb. water extract, thereby indicating that polar molecules are responsible for the enzyme inhibition (Table 1) (Lau et al., 2008). Furthermore, compounds isolated from *I. indigotica* Fortune ex Lindl. (a member of broccoli family) such as sinigrin, beta-sitosterol, and indigo (Figure 2) significantly inhibited cleavage activities of the 3CL^pro^ (Figure 1) (Lin et al., 2005). Sinigrin with an IC_50_ of 217 µM was found more effective than indigo compound (IC_50_: 752 µM) or beta-sitosterol (IC_50_: 1,210 µM) in inhibiting the cleavage processing of the 3CL^pro^ in a cell-based assay (Table 1) (Lin et al., 2005). In addition, aloe emodin and hesperetin, which are phenolic compounds from *I. indigotica* Fortune ex Lindl. (Figure 2), also dose-dependently inhibited the cleavage activity of 3CL^pro^, in which the IC_50_ for aloe emodin was 366 and 8.3 µM for hesperetin (Table 1) (Lin et al., 2005). Moreover, *R. palmatum* L. ethyl acetate extract also had anti-SARS-3CL^pro^ activity with the IC_50_ being 13.76 μg/ml (Table 2) and the level of inhibition being up to 96% (Luo et al., 2009). This high rate of 3CL^pro^ inhibitory activity from different plants suggests that these extracts or isolated compounds may represent a potential therapeutic agent against coronavirus.

### Inhibition of Viral Release and Enhancement of Host Immunity

Plant compounds also have been identified to specifically target the proteins involved in the release mechanism of the virus such as 3a ion-channel proteins (Figure 1) (Krüger and Fischer, 2009; Fischer et al., 2010; Wang et al., 2011; Kelly et al., 2003; Montal, 2003; Lu et al., 2006). Thereby, herbal medicine that suppressed the channel protein could thus be anticipated to prevent the viral spread to other cells (Schwarz et al., 2012). Employment of the anthraquinone emodin that has been used as alternative therapy in treatment of SARS has demonstrated that it can block the 3a ion channel, thus inhibiting the release of virus at concentration of 20 μM (Figures 1, 2 and Table 1) (Schwarz et al., 2012). Besides that, the flavonols, kaempferol and kaempferol glycosides (Figure 2) also showed potent inhibition towards the 3a channels (Table 1) (Schwarz et al., 2012), which suggests their potential role in inhibiting coronavirus release.

Another strategy to fight against the viral infection is by boosting the host immunity (Lau et al., 2008; Cho et al., 2012). The host with better immunity may act as physiological resistance to protect itself from any infection such as increase in the formation of white blood cells which are able to destroy the viruses rapidly. Evidently, the *in vivo* study of *Epimedium koreanum* Nakai on PEDV showed this herbal treatment protects against disease symptoms such as treating diarrhea and ensuring clean intestine in pigs (Table 3) (Cho et al., 2012). It has been suggested that the antiviral effect of *E. koreanum* Nakai is modulated by immune responses including macrophage and lymphocyte stimulation (Cho et al., 2012). Quercetin and icariin are the main compounds of *E. koreanum* Nakai (Cho et al., 2012), and interestingly, a previous study has identified that the quercetin also may be able to inhibit the replication of PEDV through specific viral induced reactive oxygen species pathway (Song et al., 2011). Besides that, an *in vitro* study of this herb also showed that this plant exhibited antiviral effect against other coronavirus subtypes such as SM98 and transmissible gastroenteritis coronavirus (TGE) viruses (Table 1) (Cho et al., 2012). This extract is not toxic within the host cells, hence indicating that it can be safely delivered for possible infection treatment (Cho et al., 2012). Besides that, *H. cordata* Thunb. study on mice has identified that the water extract from this plant was able to increase immune system of the mice through significantly stimulating the proliferation of mouse spleen lymphocytes (Lau et al., 2008). It was also revealed that *H. cordata* Thunb. increased the CD4 + and CD8 + T cell proportion and also caused a significant increment of mouse splenic lymphocyte secretion of interleukin-2 (IL-2) and interleukin-10 (IL-10) (Lau et al., 2008). These findings demonstrated that *H. cordata* Thunb. extract could exhibit immunostimulatory effect which may aid to suppress the coronavirus infection. Various other studies have also shown that diets including micronutrients such as vitamin C and D have the potential to prevent or treat COVID-19 by fortifying immune system, some of which have entered clinical trial phase (Di Matteo et al., 2020).

### Other Mechanisms

There are also other studies that have provided excellent evidence regarding natural herbs and traditional medicine as future anti-coronavirus compounds, even though its exact mechanism is still unknown. For instance, plants such as *Euphorbia neriifolia* L. and *Pelargonium sidoides* DC. were identified as having appreciable antiviral activity against human coronavirus infection. Several compounds such as 3β-friedelanol, 3β-acetoxy friedelane, friedelin, and epitaraxerol (Figure 2) that successfully isolated from the ethanolic extract of *E. neriifolia* L. leaves exhibited potent anti-human coronavirus (HCoV) activity on human fibroblasts (MRC-5) infected cells, as opposed to the positive control, actinomycin D (Table 2) (Chang et al., 2012). Structure-activity relationship further demonstrated the importance of friedelane skeleton for developing a new anti-HCoV-229E treatment (Chang et al., 2012). Moreover, *Pelargonium sidoides* DC. extract EPs® 7630 that is approved for treating acute bronchitis has broad spectrum antiviral agent activity toward various respiratory viruses including human coronavirus (Table 1) (Michaelis et al., 2011). Infected cells treated with the extract showed high survivability which was up to 87% (Table 1) (Michaelis et al., 2011). Besides that, the *P. sidoides* DC. roots have been used for generations in Southern Africa for the medication of different diseases including airways infections (Conrad et al., 2007; Brendler and van Wyk, 2008; Kolodziej, 2008) and in 2005, the Federal Institute of Drugs and Medical Devices approved its standardized extract for the use of acute bronchitis in Germany (Conrad et al., 2007). However, more work may be needed to establish the extract efficacy in treating the coronavirus infection.

*Lycoris radiata* (L'Hér.) Herb. extract also was identified as having potent anti-coronovirus activity based on a screening through an *in vitro* study (Li et al., 2005). Ethanol extract of *L. radiata* (L'Hér.) Herb. that has been treated on SARS-CoV BJ-006 strain showed the highest SI (370-422) compared with the other plants such as *Lindera aggregata* (Sims) Kosterm. (SI: 16-17), *Pyrrosia lingua* (Thunb.) Farw. (SI: 55-59), and *Artemisia annua* L. (SI: 27-31) (Table 1) (Li et al., 2005). Further structure and activity analysis resulted in a single bioactive compound called lycorine being identified as an active component of anti-SARS-CoV in *L. radiata* (L'Hér.) Herb. with an IC_*50*_ value of 15.7 nM (Table 1 and Figure 2). This compound also has a CC_50_ value of 14,980 nM in cytotoxicity assay and a selective index (SI) greater than 900 (Table 1) (Li et al., 2005). This finding has shown that lycorine may serve as a great candidate for the new anti-coronavirus treatment (Li et al., 2005).

Besides that, other plants such as *Toona sinensis* (Juss.) M. Roem. (Chinese mahogany) have been identified to possess antiviral activity toward coronavirus infection too. *T. sinensis* (Juss.) M. Roem (also known as *Cedrela sinensis*, belongs to the family Meliacceae) is popular in Taiwan, China, and Malaysia, has also been tested for SARS-CoV inhibition *in vitro* analysis and the water extract was shown to inhibit SARS coronavirus replication with an SI of 17 (Table 2) (Chen et al., 2008). However, the main compound responsible for inhibiting the SARS-CoV is still unknown, although many bioactive compounds having been isolated from its leaves including beta-sitosterol, beta-sitosteryl-glucoside, (+)-catechin, (−)-epicatechin, gallic acid, kaempferol, kaempferol-d-glucoside, methyl gallate, phytol, quercetin, quercitrin, rutin, stigmasterol, stigmasterol glucoside and toosendanin (Chia, 2007).

Additionally, *in vivo* study of chicken embryo that received treatment from garlic extract (*Allium sativum* L*.*) showed inhibitory effects against IBV, the coronavirus that contributes to tremendous economic loss in the poultry industry around the world (Mohajer Shojai et al., 2016). These treated embryos showed less dwarfism compared to the untreated group (Table 3) (Mohajer Shojai et al., 2016). The metabolite profiling of this extract also showed highly abundant compounds such as diallyl disulphide, disulfide di-2-propenyl and methyl 2 propenyl disulphide that might be responsible for its bioactivity (Mohajer Shojai et al., 2016). Furthermore, *H. cordata* Thunb. water extracts have been shown to have an inhibitory effect on the IBV (Table 3) (Yin et al., 2011). *In vitro* and *in vivo* study of *H. cordata* Thunb. conducted on specific pathogen free (SPF) chicken embryos and chickens demonstrated inhibitory activity of more than 90% against IBV infection in Vero cells and chicken embryo kidney cells as well as reduction of more than 90% of apoptosis-inducing cell death resulting from IBV infection (Yin et al., 2011). Besides that, *H. cordata* Thunb. also protected chickens against the virus during pre-treatment (62.5 mg/ml extract given before IBV infection) or post-treatment (62.5 mg/ml given after IBV infection), curing more than 50% of the infected chickens (Tables 2 and 3) (Yin et al., 2011). Additionally, ethyl acetate fraction of *H. cordata* Thunb. demonstrated anti-MHV activity at an IC_50_ of 0.98 μg/ml (Table 1) without any appreciable cytotoxic effects on CCL9.1 cells (Chiow et al., 2016). The flavonoids *of H. cordata* Thunb. which is quercetin (Figure 1) also significantly inhibit MHV activity with IC_50_ of 125 μg/ml (Table 1) (Chiow et al., 2016). Interestingly, quercetin is currently being tested in a clinical trial due to its strong antioxidant and prophylactic effect against COVID-19 (Di Matteo et al., 2020). Although these evidence suggest the use of these plant extracts for anti-coronaviral treatments, more in-depth studies are needed to elucidate their exact mechanism in targeting the viral infection.

## Conclusion

A considerable amount of research strongly indicates that both excellently characterized and less familiar medicinal plants around the world, either crude extracts or bioactive compounds from these plants, are very convincing as therapy for the new emerging coronavirus infection. Several compounds such as TGG, emodin, glycyrrhizin, aurantiamide acetate, and caffeic acid have been identified to have inhibition on viral attachment and penetration. Besides that, several plants and compounds such as *Sanguisorba officinalis* L.*, Stephania tetrandra* S. Moore (tetrandrine, fangchinoline, and cepharanthine)*,* and *Strobilanthes cusia* (Nees) Kuntze (tryptanthrin), and *F. suspensa* (Thunb.) Vahl (forsythoside A) were able to inhibit the viral RNA and protein synthesis. Other compounds such as kaempferol, N-cis-feruloyltyramine, and quercetin have targeted and inhibited the viral proteases such as 3CL^pro^ and PL^pro^, important enzymes for the co-translation of non-structural proteins. We also identified a few compounds that may act on the viral release mechanism such as through the 3a ion channel (emodin and kaempferol) as well as enhancing the host immune systems (*E. koreanum* Nakai and *H. cordata* Thunb. extracts)*.* Last but not least, several plants such as *L. radiata* (L'Hér.) Herb.*, A. sativum* L.*, E. neriifolia* L., *P. sidoides* DC.*,* and *T. sinensis* (Juss.) M. Roem. have also been discovered to have anti-coronavirus activity, yet their exact mechanisms in targeting the coronavirus infection are still unknown, thus becoming exciting candidates for the development of new anti-coronavirus agents. Perhaps the most promising extracts for this current pandemic are from *L. radiata* (ethanol extract and lycorine) and *S. cusia* (methanol extract, tryptanthrin and indigodole B) and *I. indigotica* (hesperetin) with excellent *in vitro* IC50 and SI values against human coronaviruses, as well as quercetin and kaempferol from various herbs as evidenced from in silico experimentation. However, more preclinical and clinical studies are needed to justify their use and efficacy against the current SARS-CoV-2 virus. Altogether, this compilation will aid researchers or clinicians to better evaluate some targeted plant extracts and bioactive compounds for an effective potential treatment against this devastating pandemic.

## Author Contributions

JR analysed, interpreted and reviewed the research articles as well as drafting the article. WA designed the research framework and critically revised the manuscript. All authors contributed to manuscript revision, read, and approved the submitted version.

## Funding

This study was funded by UKM Research University grants, DIP-2018-001 and GP-2019-K019471.

## Conflict of Interest

The authors declare that the research was conducted in the absence of any commercial or financial relationships that could be construed as a potential conflict of interest.
